# Comparative analysis of routine peripheral blood parameters in influenza and COVID-19 patients: a retrospective study

**DOI:** 10.1590/S1678-9946202668057

**Published:** 2026-08-21

**Authors:** Mingquan Shang, Xin Ma, Qiang Xu, Hongmei Niu, Fuqian Zhao, Lei Wang, Daofu Shen

**Affiliations:** 1 Chifeng Municipal Hospital, Department of Clinical Laboratory, Chifeng, the People’s Republic of China. E-mail: shahexiang2008@163.com; 2 China Railway Construction Corporation, Beijing Tiejian Hospital, Beijing, the People’s Republic of China; 3 North China University of Science and Technology Affiliated Hospital, Equipment Management Department, Tangshan, the People’s Republic of China; 4 North China University of Science and Technology Affiliated Hospital, Precision Medicine Sequencing Center, Tangshan, the People’s Republic of China. E-mail: lncyly@126.com

**Keywords:** Influenza, COVID-19, White blood cell count, Procalcitonin, Differential diagnosis

## Abstract

Influenza and COVID-19 are major causes of respiratory illness, and differentiating between them remains a clinical challenge when molecular testing is unavailable. This retrospective study evaluated whether routine peripheral blood parameters could aid in this differentiation. Data from 848 participants (225 influenza, 358 COVID-19, and 265 healthy controls) were analyzed. Among all tested parameters, only white blood cell (WBC) count and age differed significantly between influenza and COVID-19 patients (median WBC: 8.45 versus 6.67×10^9^/L, *p*<0.001; median age: 67 versus 71 years, *p*<0.001). Notably, procalcitonin (PCT) levels did not differ significantly between the two groups (*p*>0.05). The composite index WBC/Age amplified intergroup differences more effectively than other composite indices, including WBC×PCT, which produced a weaker effect. Receiver operating characteristic analysis revealed poor discrimination for all parameters and models: WBC count (AUC=0.67), age (AUC=0.59), WBC×PCT (AUC=0.60), and a logistic regression model combining WBC and age (AUC=0.67). Age-stratified analysis confirmed that WBC count remained significantly higher in influenza patients in both younger and older subgroups, but discriminatory performance was similarly limited (AUC=0.64 and 0.68, respectively). These findings indicate that, despite statistical differences in WBC count and age, routine blood parameters-whether alone or in combination-lack sufficient discriminatory power to reliably differentiate influenza from COVID-19 in this study. Molecular testing remains essential for accurate diagnosis.

## INTRODUCTION

Influenza is an acute viral respiratory illness caused by influenza A and B viruses, leading to a significant global disease burden annually^1^. Clinical manifestations range from self-limiting upper respiratory tract infections to severe pneumonia, extrapulmonary complications, and even fatal outcomes, particularly during pandemics, when antigenic shift results in widespread susceptibility^2^. Accurate and timely diagnosis is therefore critical for appropriate clinical management and infection control.

The evolution of laboratory diagnostics for respiratory viruses has been remarkable. While traditional methods relied on viral culture and antigen detection^3^, the advent of molecular techniques, especially polymerase chain reaction (PCR), has revolutionized the field. PCR has become the gold standard for respiratory virus identification and quantification due to its high sensitivity, specificity, and ability to identify and quantify viral pathogens^4^ ^), (^ ^5^. However, its use in routine or resource-limited settings may be constrained by the need for specialized equipment and technical expertise, relatively long sample processing, and the potential for pre-analytical errors that may affect result accuracy^6^.

This gap highlights the need for complementary, rapid, and accessible diagnostic strategies. In this context, routine laboratory parameters, which are readily available, cost-effective, and routinely measured at patient admission, represent a promising alternative for initial screening or triage. The host’s peripheral blood response to infection involves complex inflammatory and immune pathways, often reflected by alterations in standard hematological and biochemical parameters^7^ ^), (^ ^8^. For instance, distinct cytokine profiles have been observed in patients with SARS-CoV-2 infection^9^. However, a comprehensive characterization of the peripheral blood parameter profile associated with influenza virus infection, particularly in comparison to other prevalent respiratory viruses such as SARS-CoV-2, remains less explored.

Furthermore, while omics technologies have identified numerous candidate biomarkers for infectious diseases^10^ ^), (^ ^11^, translating these discoveries into validated, clinically applicable single-marker tests has proven challenging. An alternative approach is the strategic integration of existing, widely available clinical parameters. Combining markers by means of mathematical operations (e.g., multiplication or ratios) may amplify the signal of a pathogenic-specific host response, potentially yielding a more robust diagnostic indicator than any single parameter alone.

Host characteristics such as age and sex are known to influence both susceptibility to and the immune response against respiratory viruses and may therefore affect peripheral blood parameters. Additionally, clinical outcomes such as length of hospital stay may differ between influenza and COVID-19 and provide ancillary information for patient management^12^ ^), (^ ^13^ ^), (^ ^14^.

Therefore, this study aims to characterize the peripheral blood parameter profiles of patients with influenza virus infection in comparison with those of patients with COVID-19 and healthy controls. We also explore sex-associated differences and age-stratified effects on laboratory parameters and compare length of hospital stay between the two infections as a secondary clinical outcome. The diagnostic value of the most promising individual and composite parameters was assessed using receiver operating characteristic analysis.

## MATERIALS AND METHODS

### Ethics

This single-center retrospective study was conducted at Chifeng Municipal Hospital. The study protocol was reviewed and approved by the hospital’s Ethics Committee. All participant data were anonymized and handled with strict confidentiality. Written informed consent was obtained from all participants.

### Study design and population

A total of 848 participants were enrolled between January and June 2023 and categorized into three groups: Influenza Group (n=225): Patients diagnosed with influenza virus infection according to the Chinese Guidelines for the Diagnosis and Treatment of Influenza (2023 Edition).

COVID-19 Group (n=358): Patients diagnosed with SARS-CoV-2 infection according to the Chinese COVID-19 Infection Diagnosis and Treatment Program (Trial Tenth Edition). This group served as a disease control to assess the specificity of the candidate indicator against another major viral respiratory pathogen.

Healthy Control Group (n=265): Individuals presenting to the outpatient clinic with respiratory symptoms who subsequently tested negative for a comprehensive panel of common respiratory pathogens. This group was intended to represent a non-infected baseline population.

### Diagnostic criteria and pathogen detection

For all participants, underlying conditions including cancer, diabetes (fasting glucose>6.1 mmol/L), severe obesity (BMI≥28 kg/m^2^), hypertension (blood pressure≥130/80 mmHg), and autoimmune diseases were exclusion criteria based on medical history and admission records. Data on age and sex were collected.

The diagnosis of influenza and COVID-19 was confirmed by specific laboratory tests:

Respiratory Pathogen Screening: Serum samples from all participants were tested for IgM antibodies against 11 common respiratory pathogens (Respiratory Syncytial Virus, Parainfluenza Virus, Adenovirus, Coxsackievirus A/B, *Mycoplasma pneumoniae*, *Chlamydia pneumoniae*, Echovirus, *Legionella pneumophila*, and Influenza A and B viruses) using an indirect immunofluorescence assay (Respiratory Pathogen Spectrum reagent, Euromon, Germany). Individuals in the Influenza Group tested positive only for Influenza Virus (A/B) IgM, and negative for all other pathogens. Individuals in the Healthy Control Group tested negative for all pathogens in this panel.

SARS-CoV-2 Detection: Pharyngeal swab samples from the COVID-19 Group were tested using a commercial SARS-CoV-2 nucleic acid detection kit (Shengxiang Bio, China), yielding a positive result. This group tested negative for influenza virus IgM.

### Data collection

For all participants, results of routine clinical laboratory tests obtained from the first blood sample collected at admission were retrospectively retrieved. The analyzed parameters included:

Hematological parameters: White blood cell (WBC) count, neutrophil percentage (N%), lymphocyte percentage (L%), monocyte percentage (Mo%), mean corpuscular volume (MCV), and red cell distribution width (RDW). Analyses were performed using a Sysmex XN series hematology analyzer (Japan).

Coagulation parameters: Prothrombin time (PT), activated partial thromboplastin time (APTT), and D-Dimer (D-D). Analyses were performed using a Sysmex Aptio coagulation analyzer (Japan).

Inflammatory biomarkers: Interleukin-6 (IL-6), procalcitonin (PCT), and C-reactive protein (CRP). IL-6 and PCT were measured on a Roche cobas 8000 automated immunoassay analyzer (Switzerland). CRP was measured using a Beckman IMMAGE 800 specific protein analyzer (USA).

### Length of hospital stay

According to the patient’s diagnosis and the physician’s judgment regarding the need for hospitalization, the length of hospital stay was determined based on the time required to achieve negative results for the peripheral blood respiratory virus spectrum and the pharyngeal coronavirus nucleic acid testing. In general, patients were discharged on the day following a negative test result. Therefore, the length of hospital stay was used to estimate the time required for viral clearance.

### Statistical analysis

Statistical analyses were performed using GraphPad Prism (version 10.0, GraphPad Software, San Diego, CA, USA) and SPSS (version 21.0, IBM Corp., Armonk, NY, USA). The normality of continuous variables was assessed within each group using the Shapiro-Wilk test. Since virtually all laboratory parameters showed significant deviation from normality (*p<*0.05 for WBC, PCT, IL-6, CRP, D-dimer, WBC×PCT, and other indices), non-parametric methods were adopted for all analyses. Continuous data are presented as medians and interquartile ranges (IQR; 25th-75th percentile). Differences among the three study groups were compared using the Kruskal-Wallis test. When the overall Kruskal-Wallis test was significant, Dunn’s post hoc test was performed for pairwise comparisons with adjustment for multiple comparisons. Two-group comparisons (e.g., sex differences within disease groups and age-stratified analyses) were performed using the Mann-Whitney U test. Receiver operating characteristic (ROC) analysis was used to evaluate the discriminatory performance of individual parameters (WBC count and age), composite indices (WBC×PCT and WBC/Age), and a multivariable logistic regression model. The area under the ROC curve (AUC) was calculated with 95% confidence intervals, and statistical significance was assessed by testing whether the AUC differed from 0.5 (no discrimination). A binary logistic regression model was constructed with influenza/COVID-19 status as the dependent variable. Variables showing significant differences in univariate analysis (WBC count and age) were entered as independent variables. The predicted probabilities generated by the model were used to generate ROC curves. Model calibration was assessed using the Hosmer-Lemeshow goodness-of-fit test. A two-tailed *p* value<0.05 was considered statistically significant for all analyses.

## RESULTS

### Baseline characteristics of the study population

The baseline demographic characteristics of the 848 enrolled participants are summarized in Supplementary Table S1. The cohort comprised 225 patients with influenza, 358 patients with COVID-19, and 265 healthy controls. Overall, 475 participants (56.0%) were male and 373 (44.0%) were female. A total of 216 individuals (25.5%) were younger than 60 years, whereas 632 (74.5%) were aged 60 years or older. All participants met the predefined exclusion criteria and had no underlying comorbidities such as cancer, diabetes, severe obesity, hypertension, or autoimmune diseases.

### Individual laboratory parameters and composite diagnostic index

After non-parametric reanalysis, only WBC count and age showed statistically significant differences between influenza and COVID-19 patients. Influenza patients had significantly higher WBC counts than both COVID-19 patients and healthy controls (both *p*<0.001). Patients with influenza were also significantly younger than those with COVID-19 (*p<*0.001). Table 1 summarizes the comparisons of all tested laboratory parameters. In contrast, no significant differences were observed between the influenza and COVID-19 groups in N%, L%, Mo%, MCV, RDW, PT, APTT, D-dimer, PCT, IL-6, or CRP (Table 1).

**Table 1:** Changes in clinical test indicators after influenza and COVID-19 infection.

Peripheral blood and demographic parameters	Healthy (n=265)	Influenza (n=225)	COVID-19 (n=358)	***p* value**
WBC count (×109/L)	6.85 (5.06-8.94)	8.45 (6.28-11.44)	6.67 (4.70-8.75)	<0.001*
N%	75.80 (66.68-84.58)	74.90 (63.10-82.65)	73.30 (63.85-82.33)	>0.99
L%	14.50(8.40-21.55)	14.5 (8.60-24.05)	15.40 (9.85-24.03)	>0.99
Mo%	7.20 (4.70-10.40)	7.40 (5.65-9.25)	8.00 (5.40-10.80)	0.174
MCV (fl)	90.10(87.73-93.08)	91.30(88.05-93.90)	91.10(88.10-94.20)	>0.99
RDW (%)	12.60(12.10-13.40)	13.00(12.40-13.75)	12.80(12.20-13.40)	0.133
PT (s)	11.70(11.20-12.70)	12.00(11.50-12.90)	11.90(11.20-12.70)	0.075
APTT (s)	26.65(24.50-29.80)	26.80(24.20-29.10)	27.20 (25.00-30.20)	0.23
D-D dimer (mg/L)	0.77(0.43-1.82)	0.92(0.46-1.83)	0.78(0.42-1.70)	>0.99
IL-6 (pg/mL)	13.20(3.44-29.90)	12.29(4.04-27.69)	11.10(3.295-32.96)	>0.99
PCT (μg/L)	0.066(0.036-0.121)	0.07(0.039-0.297)	0.065(0.043-0.123)	0.539
CRP (mg/L)	22.90(10.98-73.90)	28.40(11.90-74.40)	20.50(9.05-62.45)	0.46
Age	68.00(60.00-76.00)	67.00(55.00-75.00)	71.00(61.00-79.00)	<0.001*

*p* values derived from Dunn’s post hoc test following significant Kruskal-Wallis test. *Bold values indicate statistical significance (*p*<0.05). All variables deviated significantly from normality (Shapiro-Wilk, *p*<0.05), justifying the use of non-parametric tests. *p* value represents the comparison between COVID-19 and influenza infection.

**Figure 1:**
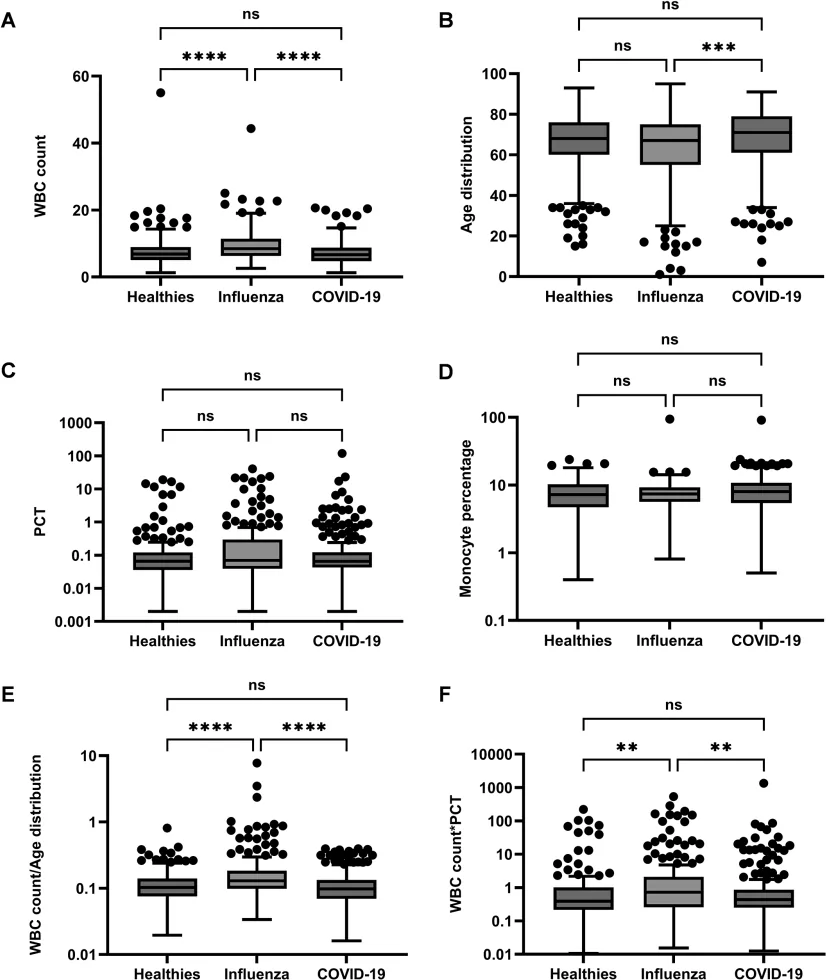
Comparison of composite indices among healthy controls, influenza, and COVID-19 groups. Peripheral blood samples were collected from healthy controls (n=265), influenza patients (n=225), and COVID-19 patients (n=358) at initial admission. Composite indices were calculated and compared using the Kruskal-Wallis test followed by Dunn’s post hoc test. Data are presented as box plots showing medians and interquartile ranges: (A) WBC count; (B) Age; (C) PCT; (D) Monocyte percentage; (E) WBC/Age; (F) WBC×PCT index. WBC count was significantly higher in the influenza group compared to both healthy controls and COVID-19 patients (both *p*<0.0001, Dunn’s test). Age was only markedly elevated in the influenza group compared to COVID-19 patients (*p*<0.001). WBC/Age index was significantly higher in influenza group than in the other two groups (both *p*<0.0001). WBC×PCT index was also significantly higher in the influenza group than in the other two groups (both *p<*0.01), but showed a smaller effect size. All other composite indices showed no significant differences among the three groups. ****p<0.0001; ***p<0.001; **p<0.01; *p<0.05; ns, not significant.

The distributions of the selected individual parameters and composite indices, including those with statistically significant differences, are illustrated in Figure 1. Influenza patients exhibited significantly higher WBC counts than both COVID-19 patients and healthy controls (Figure 1A; both *p<*0.0001 by Dunn’s post hoc test following the Kruskal-Wallis test). Age was only significantly lower in the influenza group than in the COVID-19 group (Figure 1B; both *p<*0.001). In contrast, procalcitonin (PCT), which had been hypothesized to be a discriminatory marker, did not differ significantly between influenza and COVID-19 patients (Figure 1C; *p*>0.05), Similarly, Mo% showed no significant differences among the three groups (Figure 1D; *p>*0.05). The composite index WBC/Age, calculated by dividing WBC count by age, was also significantly higher in influenza patients compared with both COVID-19 patients and healthy controls (Figure 1E; both *p*<0.0001). The WBC×PCT index, which had been proposed as a potential discriminatory marker, also differed significantly between influenza and COVID-19 patients (Figure 1F; *p*<0.01). Notably, WBC/Age yielded stronger statistical significance than all other composite indices.

### Analysis of sex-associated differences

We further investigated potential sex-related differences in laboratory parameters within the COVID-19 and influenza groups.

Among patients with COVID-19, several parameters showed significant sex disparity (Table 2). Males had significantly higher WBC counts, N%, PT, APTT, PCT, and CRP levels (*p<*0.05), while L% was significantly lower compared to females (*p*<0.001). No significant sex differences were found for Mo%, MCV, RDW, D-dimer, IL-6, or age in the COVID-19 group.

**Table 2:** Changes in clinical test indicators of different genders after COVID-19 infection.

Peripheral blood and demographic parameters	Man (COVID-19) n=207	Woman (COVID-19) n=151	***p* value (Man vs. Woman)**
WBC count (×109/L)	6.86 (4.89-8.83)	6.14 (4.42-8.50)	0.021*
N%	74.43 (66.20-82.70)	72.70 (61. 35-81.35)	0.043*
L%	14.05 (8.80-21.25)	18.00 (10.93-27.08)	0.001*
Mo%	8.10 (5.38-11.05)	7.90 (5.55-10.35)	0.733
MCV (fl)	91.30 (88.10-94.30)	90.60 (87.90-94.15)	0.592
RDW (%)	12.90 (12.20-13.63)	12.80 (12.13-13.38)	0.46
PT (s)	12.16 (11.40-12.80)	11.60 (10.90-12.40)	0.0001*
APTT (s)	28.25 (25.48-30.80)	26.20 (24.00-29.20)	<0.001*
D-D dimer (mg/L)	0.85 (0.39-1.90)	0.72 (0.52-1.09)	0.307
IL-6 (pg/mL)	13.23 (4.02-34.16)	9.34 (3.23-30.01)	0.283
PCT (μg/L)	0.071 (0.047-0.152)	0.054 (0.037-0.087)	0.007*
CRP (mg/L)	27.30 (12.60-79.00)	13.65 (6.56-36.18)	<0.001*
Age	72.00(61.00-80.00)	71.00(61.00-79.00)	0.8885

Data are median (IQR). *p* values are derived from the Mann-Whitney U test. *Bold: *p*<0.05.

In contrast, patients with influenza showed fewer sex disparities. Only PT, APTT, PCT, and CRP levels were significantly higher in males (*p*<0.05), while WBC count, N%, L %, Mo%, MCV, RDW, D-dimer, IL-6, and age did not differ between sexes (Table 3).

**Table 3:** Changes of clinical test indicators in different genders after influenza infection.

Peripheral blood and demographic parameters	Man (Influenza) n=127	Woman (Influenza) n=98	***p* value (Man vs. Woman)**
WBC count (×109/L)	8.80 (6.54-11.53)	7.92 (5.91-11.28)	0.206
N%	74.80 (63.85-83.13)	75.00 (61.70-81.80)	0.323
L%	13.70 (7.83-22.83)	15.30 (8.70-25.45)	0.322
Mo%	7.10 (5.25-9.20)	7.50 (6.10-9.60)	0.417
MCV (fl)	91.90 (88.70-93.98)	91.10 (87.70-93.85)	0.427
RDW (%)	13.05 (12.40-14.20)	12.60 (12.20-13.40)	0.083
PT (s)	12.30 (11.70-13.53)	11.70 (11.20-12.50)	<0.001*
APTT (s)	27.60 (25.10-29.80)	25.90(23.60-28.20)	0.006*
D-D dimer (mg/L)	1.04 (0.49-1.59)	0.85 (0.27-2.02)	0.665
IL-6 (pg/mL)	15.16 (4.17-33.23)	6.85 (2.94-15.34)	0.085
PCT (μg/L)	0.100 (0.052-0.497)	0.048 (0.028-0.123)	<0.001*
CRP (mg/L)	41.20 (16.40-82.00)	18.20 (6.04-39.10)	<0.001*
Age	65.00(55.00-75.00)	68.00(58.00-77.00)	0.451

Data are medians (IQR). *p* values are derived from the Mann-Whitney U test. *Bold: *p*<0.05.

### ROC analysis of discriminatory performance

**Figure 2:**
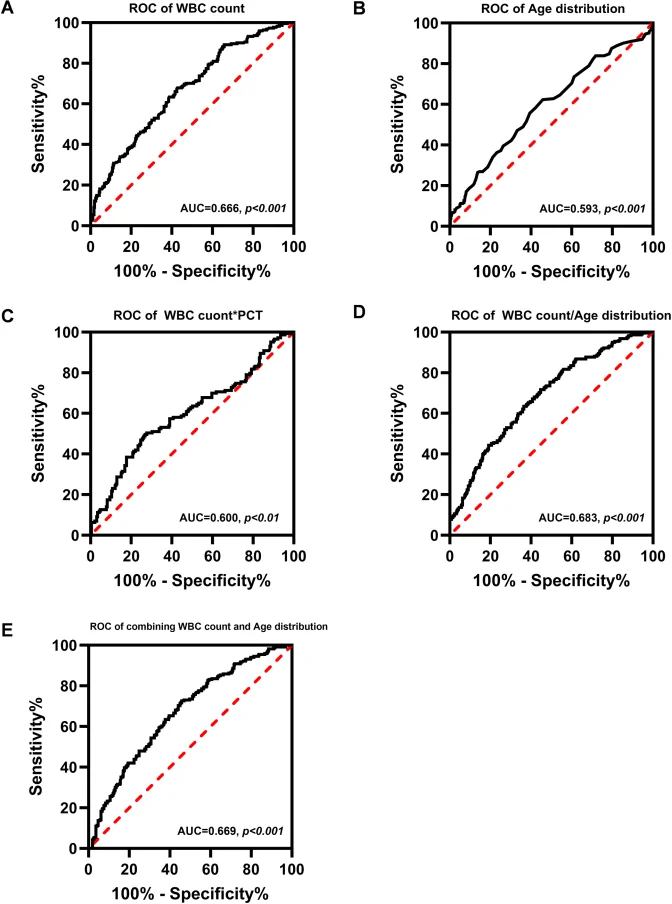
ROC curves for individual parameters, composite indices, and a multivariable model for discriminating influenza from COVID-19. ROC curves were constructed to evaluate the discriminatory performance of the following: (A) WBC count alone (AUC=0.666; *p<*0.001); (B) age alone (AUC=0.593; *p*<0.001); (C) the product of WBC count and procalcitonin (WBC×PCT; AUC=0.600; *p*<0.01); (D) the WBC/Age index, calculated as the quotient of WBC count divided by age (AUC=0.683; *p*<0.001); and (E) the predicted probability from a binary logistic regression model incorporating WBC count and age as independent variables (combined model; AUC=0.669; *p*<0.001). Although all AUCs were significantly greater than 0.5, indicating better-than-chance discrimination, none reached the threshold of 0.70 generally considered minimally acceptable for clinical utility. The WBC/Age index and the logistic regression model showed marginally improved performance compared with individual parameters, but overall discriminatory ability remained limited. The diagonal reference line represents an AUC of 0.5 (no discrimination). All *p* values were derived from tests of the null hypothesis that AUC=0.5. ***p<0.001; **p<0.01.

ROC analysis was performed to assess the ability of individual and combined parameters to discriminate influenza from COVID-19 (Figure 2). All tested parameters and models showed limited discriminatory performance. WBC count alone yielded an AUC of 0.67 (*p*<0.001), age alone an AUC of 0.59 (*p<*0.001), WBC×PCT an AUC of 0.60 (*p*<0.01), WBC/Age an AUC of 0.68 (*p<*0.001), and the logistic regression model (WBC+Age) an AUC of 0.67 (*p<*0.001). Although all AUCs were significantly greater than 0.5, indicating discrimination better than chance, none reached the threshold of 0.70 generally considered minimally acceptable for clinical utility. The WBC/Age index showed a marginal improvement (AUC=0.68) but remained inadequate for clinical application.

### Age-stratified analysis and length of hospital stay

**Figure 3:**
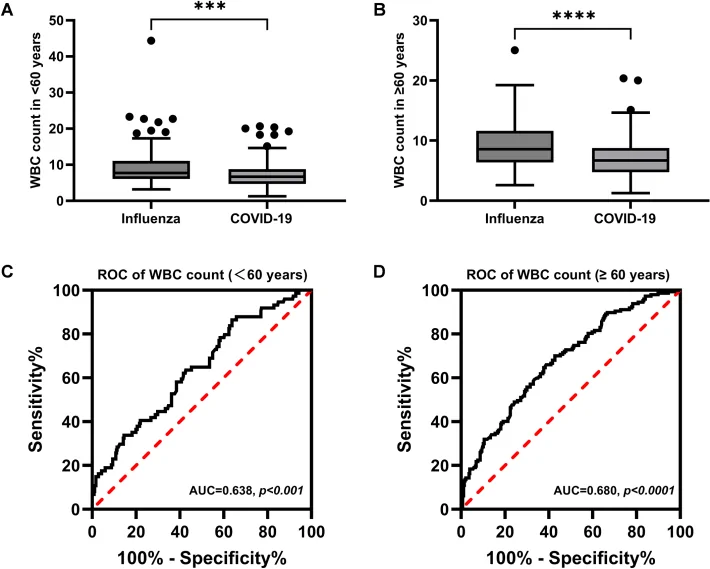
Age-stratified comparison and discriminatory performance of white blood cell (WBC) count between influenza and COVID-19 patients: (A, B) Box plots comparing WBC counts between influenza and COVID-19 patients stratified by age: (A) patients aged<60 years and (B) patients aged≥60 years. Data are presented as medians, interquartile ranges, and Tukey whiskers. Groups were compared using the Mann-Whitney U test; WBC counts were significantly higher in influenza patients in both age strata (*p<*0.001 for<60 years; *p*<0.0001 for≥60 years); (C, D) ROC curves evaluating the ability of WBC count alone to discriminate influenza from COVID-19 within each age stratum: (C) patients aged<60 years (AUC=0.638; *p*<0.001) and (D) patients aged≥60 years (AUC=0.680; *p*<0.0001). Although WBC counts differed significantly between groups in both younger and older patients, the area under the ROC curve remained below 0.70, indicating that, even after accounting for age, WBC count alone provides limited discriminatory performance for distinguishing influenza from COVID-19. The diagonal reference line denotes an AUC of 0.5 (no discrimination). ****p<0.0001; ***p<0.001.

To determine whether the difference in WBC count between influenza and COVID-19 was influenced by age, we performed age-stratified analysis (Figure 3). WBC count remained significantly higher in influenza patients in both age strata: <60 years (*p*<0.001; Figure 3A) and≥60 years (*p*<0.0001; Figure 3B). However, ROC analysis within each age stratum showed similarly limited discriminatory performance, with an AUC of 0.64 for patients <60 years and 0.68 for patients ≥60 years (Figures 3C and 3D). These findings indicate that, although the WBC difference is independent of age, its diagnostic value does not reach clinical thresholds even within homogeneous age groups.

Comparison of length of hospital stay between influenza and COVID-19 patients showed no significant overall difference (Supplementary Figure S1A). When stratified by age (<60 and≥60 years), no significant differences were observed in either subgroup (Supplementary Figures S1B and S1C). Sex-stratified analysis revealed that, among male patients, those with influenza had a significantly shorter hospital stay than those with COVID-19 (*p*<0.05; Supplementary Figure S1D), while no significant difference was found among female patients (Supplementary Figure S1E). Within the COVID-19 group, male patients had significantly longer hospital stays than female patients (*p*<0.001; Supplementary Figure S1F).

## DISCUSSION

This study evaluated whether routine peripheral blood parameters could discriminate influenza from COVID-19 in a cohort of 848 participants. After rigorous normality testing and the application of non-parametric methods, the principal findings are twofold. First, among a broad range of hematological, inflammatory, and coagulation parameters, only WBC count and age differed significantly between the two infections. Second, and more importantly, the discriminatory performance of these parameters-whether assessed individually, as composite indices, or within a multivariable model-was consistently poor, with all areas under the ROC curve (AUC) remaining below 0.70.

The significantly higher WBC count in influenza patients is consistent with the well-established capacity of influenza viruses to elicit peripheral blood leukocytosis, driven by neutrophilic mobilization and systemic inflammatory mediators^15^ ^), (^ ^16^ ^), (^ ^17^. In contrast, SARS-CoV-2 infection is more frequently associated with a normal or decreased WBC count and lymphopenia^7^ ^), (^ ^9^. The present findings reinforce this distinction at the group level; however, yet the substantial overlap in WBC distributions between the two diseases limits its clinical applicability. The younger age of influenza patients in our cohort likely reflects local epidemiology, and age-stratified analysis confirmed that the WBC difference persisted independently of age. The composite index WBC/Age, which combined these two variables, yielded a slightly higher AUC (0.68) than either parameter alone, but still did not reach a clinically useful threshold.

A noteworthy observation is that PCT did not differ significantly between influenza and COVID-19. Although PCT has been proposed as a potential discriminator in some comparative studies^18^ ^), (^ ^19^, this non-parametric analysis found no significant difference (*p*>0.05). This likely reflects the modest and inconsistent PCT response that may occur in severe viral infections, irrespective of etiology^20^ ^), (^ ^21^. Moreover, in the absence of comprehensive microbiological testing for bacterial co-infection, isolated PCT elevations cannot be reliably attributed to a specific virus^22^ ^), (^ ^23^. Thus, PCT does not appear to offer meaningful discriminatory value in this context.

Other parameters, including Mo%, CRP, D-dimer, and coagulation indices, also failed to differentiate between the two infections. These findings suggest that the systemic inflammatory and coagulopathic responses triggered by influenza and SARS-CoV-2, while potentially distinct in magnitude or kinetics, are not captured by single time-point routine blood tests in a manner that enables reliable discrimination^7^ ^), (^ ^8^. We hypothesized that composite indices, such as WBC×PCT, could enhance disease-specific discriminatory signals. However, only WBC/Age, derived from the two individually significant parameters, achieved relatively better performance. All other composite indices were less effective, with AUC values consistently below 0.70. These findings indicate that basic mathematical combinations of routine laboratory tests have limited practical value for differentiating the two viral infections.

The consistently poor AUC values-ranging from 0.59 for age alone to 0.68 for the WBC/Age interaction and the logistic regression model-deserve particular attention. Although all AUCs were statistically greater than 0.5 (*p*<0.001 for most), this merely indicates better-than-chance discrimination, which is a minimal requirement. A clinically useful diagnostic test generally requires an AUC of at least 0.80, whereas values below 0.70 are considered inadequate^24^. The marked discrepancy between the significant p-values from non-parametric group comparisons and the low AUCs highlights a critical principle: statistical significance does not necessarily translate into clinical usefulness, especially when the distributions of measured parameters show extensive overlap^25^.

Age-stratified analysis provided further insight. The WBC difference between influenza and COVID-19 persisted in both younger and older patients, but the AUCs within each stratum (0.64 for<60 years, 0.68 for≥60 years) were similarly limited. The modest improvement observed in older patients may be related to the higher overall inflammatory burden in this group^15^ ^), (^ ^16^, yet it remains insufficient for diagnostic application. These results indicate that the discriminative information provided by WBC count and age is statistically detectable but too weak to guide clinical decision-making at the individual patient level.

Gender-stratified analyses revealed distinct immunoinflammatory profiles. COVID-19 patients showed pronounced sex-related differences: males exhibited higher WBC counts, neutrophil percentage, PT, and CRP levels, as well as lower lymphocyte percentages, a pattern consistent with the more severe disease course observed in males^26^. In contrast, influenza patients displayed fewer sex-related disparities, with only PT, APTT, PCT, and CRP levels differing significantly. This relative homogeneity in influenza suggests a more uniform host response across sexes, potentially reflecting fundamental differences in the immunopathogenesis of the two viruses. These exploratory findings may inform future studies on sex-specific host responses.

Length of hospital stay did not differ significantly between influenza and COVID-19 overall, mirroring the clinical overlap that makes differential diagnosis challenging. The observation that male COVID-19 patients had longer hospital stays than female COVID-19 patients is consistent with prior reports^26^, while the shorter hospital stay observed in male influenza patients compared with male COVID-19 patients may reflect the differing severity profiles of the two illnesses among men.

Several limitations should be considered. First, the retrospective, single-center design may affect generalizability. Second, although the sample size was substantial, the study may have been underpowered to detect very small effect sizes. Third, and importantly, the absence of systematic bacterial co-infection testing means we cannot exclude the possibility that undetected bacterial infections influenced WBC and PCT levels^22^ ^), (^ ^23^. Although the lack of significant PCT differences between influenza and COVID-19 partially mitigates this concern, future studies should incorporate comprehensive microbiological assessments. Fourth, interleukin-6 data were available for only a small subset of participants and were therefore excluded from primary analyses to avoid selection bias. Fifth, we did not perform serial measurements, which could have revealed time-dependent differences. Finally, the study lacked data on other respiratory viruses, such as respiratory syncytial virus and adenovirus, limiting the assessment of specificity.

## CONCLUSION

This study demonstrates that, among routine peripheral blood parameters, only WBC count and age differed statistically between influenza and COVID-19, but these differences were insufficient for clinical discrimination. No single parameter, composite index, or multivariable model achieved a clinically meaningful AUC. These findings do not support the use of routine blood tests as standalone tools for differentiating these two viral infections, and molecular testing remains essential for accurate diagnosis. Future efforts should focus on identifying novel pathogen-specific biomarkers and should include rigorous assessment for bacterial co-infections.

## Data Availability

The complete anonymized dataset supporting the findings of this study is available from https://doi.org/10.48331/SCIELODATA.U9YM5D

## SUPPLEMENTARY MATERIAL

Supplementary Table S1 Baseline and clinical characteristics in patients.

Supplementary Figure S1 Comparison of length of hospital stay between influenza and COVID-19 patients, overall and stratified by age and sex: (A) Overall comparison of length of hospital stay between all influenza and COVID-19 patients showed no significant difference (Mann-Whitney U test); (B, C) When stratified by age, no significant difference was observed in either patients aged < 60 years (B) or those aged ≥ 60 years (C); (D, E) When stratified by sex, length of stay was significantly shorter in male influenza patients than in male COVID-19 patients (*p* < 0.05; (D), whereas no significant difference was found among female patients (E); (F) Within the COVID-19 group, male patients had a significantly longer hospital stay compared with female patients (*p* < 0.001). No other subgroup comparisons reached statistical significance. Data are presented as box plots (medians, interquartile ranges, Tukey whiskers). Groups were compared using the Mann-Whitney U test. These results indicate that length of hospital stay did not reliably differ between influenza and COVID-19 overall, although sex-specific differences may exist in certain subgroups. ****p* < 0.001; **p* < 0.05; ns, not significant.
