# Supplementary material for: Comparative analysis of routine peripheral blood parameters in influenza and COVID-19 patients: a retrospective study
Source: Rev Inst Med Trop Sao Paulo. 2026 Aug 21;68:e57. doi: 10.1590/S1678-9946202668057 (PMC13502944; doi:10.1590/S1678-9946202668057)
Supplement: Supplementary file 1 [file 1678-9946-rimtsp-68-S1678-9946202668057-ms1.pdf]

REVISTA DO INSTITUTO DE MEDICINA TROPICAL DE SÃO PAULO

JOURNAL OF THE SÃO PAULO INSTITUTE OF TROPICAL MEDICINE

SUPPLEMENTARY MATERIAL

https://doi.org/10.48331/SCIELODATA.U9YM5D

Comparative analysis of routine peripheral blood parameters in influenza and COVID-19 patients: a retrospective study

Mingquan Shang[1*], Xin Ma[2*], Qiang Xu[3*], Hongmei Niu[1], Fuqian Zhao[1], Lei Wang[4], Daofu Shen[1]

[1]Chifeng Municipal Hospital, Department of Clinical Laboratory, Chifeng, the People's Republic of China

[2]China Railway Construction Corporation, Beijing Tiejian Hospital, Beijing, the People's Republic of China

[3]North China University of Science and Technology Affiliated Hospital, Equipment Management Department, Tangshan, the People's Republic of China

[4]North China University of Science and Technology Affiliated Hospital, Precision Medicine Sequencing Center, Tangshan, the People's Republic of China

*These authors contributed equally to the article

Correspondence to: Daofu Shen
Chifeng Municipal Hospital, Department of Clinical Laboratory, No.152 Changqing Street, Hongshan District, Chifeng City, Inner Mongolia Autonomous Region, the People's Republic of China
E-mail: shahexiang2008@163.com

Lei Wang
North China University of Science and Technology Affiliated Hospital, Precision Medicine Sequencing Center, No.73 South Jianshe Road, Lubei District, Tangshan City, Hebei Province, the People's Republic of China
E-mail: lncyly@126.com

Received: 6 March 2026
Accepted: 22 June 2026

Editor: Camila Malta Romano

Supplementary Table S1 - Baseline and clinical characteristics in patients.

Characteristics	Young patients (< 60 years) (n = 216)		Old patients (≥ 60 years)(n = 632)		All patients (n = 848)	
	N	%	N	%	N	%
**Sex**						
Man	127	15.0%	348	41.0%	475	56.0%
Woman	89	10.5%	284	33.5%	373	44.0%
**Group**						
Healthy	62	7.3%	203	23.9%	265	31.3%
Influenza	78	9.2%	147	17.3%	225	26.5%
COVID-19	76	9.0%	282	33.3%	358	42.2%
**Age**						
< 19 years	15.00 (6.25-16.25)					
19–35years	29.00 (25.00-34.00)					
36–59 years	55.00 (50.00-58.00)					
≥ 60years	73.00 (68.00-80.00)					

Percent values (%) were calculated as a percentage of the column total.

This is an open-access article distributed under the terms of the Creative Commons Attribution License.

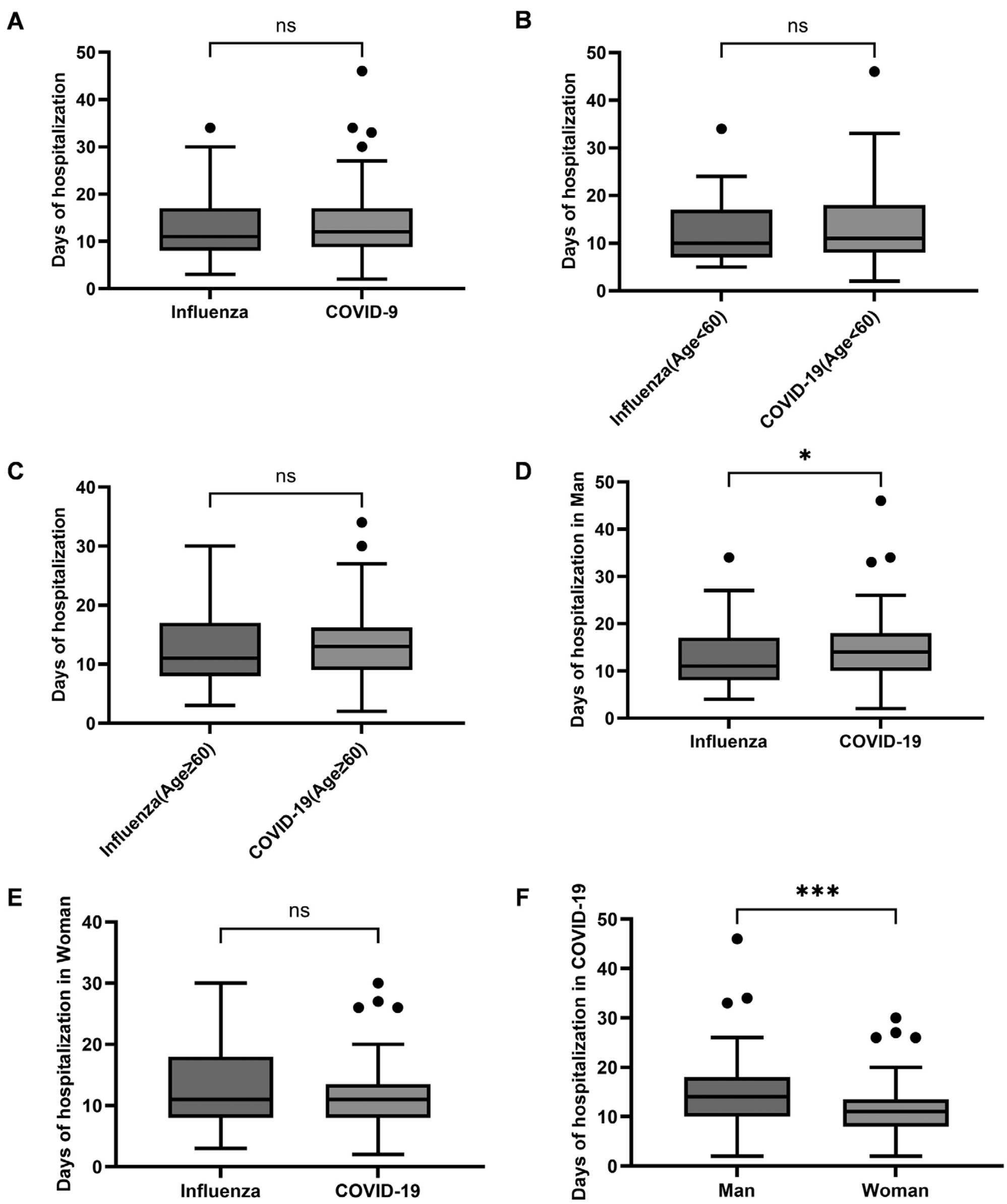

Supplementary Figure S1 - Comparison of length of hospital stay between influenza and COVID-19 patients, overall and stratified by age and sex: (A) Overall comparison of length of hospital stay between all influenza and COVID-19 patients showed no significant difference (Mann-Whitney U test); (B, C) When stratified by age, no significant difference was observed in either patients aged < 60 years (B) or those aged ≥ 60 years (C); (D, E) When stratified by sex, length of stay was significantly shorter in male influenza patients than in male COVID-19 patients ($p < 0.05$; (D), whereas no significant difference was found among female patients (E); (F) Within the COVID-19 group, male patients had a significantly longer hospital stay compared with female patients ($p < 0.001$). No other subgroup comparisons reached statistical significance. Data are presented as box plots (medians, interquartile ranges, Tukey whiskers). Groups were compared using the Mann-Whitney U test. These results indicate that length of hospital stay did not reliably differ between influenza and COVID-19 overall, although sex-specific differences may exist in certain subgroups. ***$p < 0.001$; *$p < 0.05$; ns, not significant.
